# Glymphatic Dysfunction Is Related to Comorbidity of Parkinson's Disease and Anxiety: A Multimodal MRI Study

**DOI:** 10.1002/brb3.70918

**Published:** 2025-09-21

**Authors:** Kaidong Chen, Liujia Lu, Li Zhang, Yi Ji, Bin He, Ruixuan Zhang, Feng Wang, Xiaoyun Hu, Xiangming Fang

**Affiliations:** ^1^ Department of Radiology The Affiliated Wuxi People's Hospital of Nanjing Medical University Wuxi China; ^2^ Department of Neurology The Affiliated Wuxi People's Hospital of Nanjing Medical University Wuxi China

**Keywords:** ALPS, anxiety, gBOLD‐CSF coupling, glymphatic system, neuroimaging biomarkers, Parkinson's disease

## Abstract

**Aims:**

This study aimed to explore the relationship between glymphatic dysfunction and anxiety in patients with Parkinson's disease (PD).

**Methods:**

A total of 21 patients with PD with anxiety (PD‐A), 47 patients with PD without anxiety (PD‐NA), and 38 healthy subjects were prospectively enrolled. Participants underwent neuropsychiatric assessment, motor examinations, and magnetic resonance imaging. Brain glymphatic function was evaluated using diffusion tensor image analysis along the perivascular spaces (DTI‐ALPS) and global blood oxygen level‐dependent signal and cerebrospinal fluid dynamics coupling (gBOLD‐CSF coupling). Correlations between glymphatic function and anxiety (quantified using Hamilton Anxiety Rating Scale [HAMA] scores) were analyzed. Receiver operating characteristic analysis was used to assess the diagnostic performance of clinical features, ALPS index, and gBOLD‐CSF coupling strength, and multivariate models were used to distinguish patients with PD‐A from those with PD‐NA.

**Results:**

The PD‐A group exhibited longer PD duration, higher Hoehn and Yahr (H&Y) stage, and higher HAMA scores compared to the PD‐NA group. Both DTI‐ALPS and gBOLD‐CSF coupling analyses revealed significant glymphatic dysfunction in patients with PD‐A. The ALPS index and gBOLD‐CSF coupling strength were inversely correlated with HAMA scores and positively correlated with each other. The multivariate diagnostic model combining clinical variables and glymphatic indices outperformed univariate models.

**Conclusions:**

The DTI‐ALPS index and gBOLD‐CSF coupling strength may serve as novel neuroimaging markers for PD‐A, with diagnostic performance enhanced by combining these markers with clinical features.

## Graphical abstract

Based on DTI‐ALPS and gBOLD‐CSF coupling analyses, we found glymphatic dysfunction may be one of the reasons for the high comorbidity of Parkinson's disease (PD) and anxiety. The diagnostic model combining glymphatic index with clinical features could significantly enhance diagnostic performance for anxiety in PD.

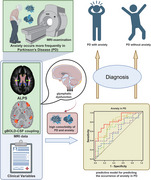

## 1 Introduction

Parkinson's disease (PD) is one of the most common neurodegenerative disorders characterized by motor symptoms (such as bradykinesia, tremor, and rigidity) and various non‐motor symptoms (Armstrong and Okun 2020). Among the non‐motor symptoms of PD, neuropsychiatric manifestations, especially anxiety, significantly impact patients’ quality of life (Ray and Agarwal 2020). Compared to other neurodegenerative diseases, anxiety occurs more frequently in patients with PD (Martínez‐Martín and Damián 2010).

The glymphatic system, often referred to as the brain's lymphatic drainage system, facilitates the clearance of large molecules and proteins from cerebrospinal fluid (CSF) circulation (Fang et al. 2020; Sun et al. 2018). Evidence suggests that dysfunction of the glymphatic system may contribute to PD pathogenesis by impairing alpha‐synuclein (α‐syn) clearance. Blocking meningeal lymphatic drainage in A53T α‐syn transgenic mice accelerates the accumulation of α‐syn, promoting the progression of PD (Zou et al. 2019). Moreover, glymphatic function decline had been observed during all stages of PD (Si et al. 2022; Zhu et al. 2025, Nepozitek et al. 2025; Marecek et al. 2025; Cai et al. 2023). As the disease advances, non‐motor symptoms such as sleep disturbance and cognitive impairment have been closely associated with reduced glymphatic clearance in PD (Si et al. 2022; Shen et al. 2022). These findings suggest that glymphatic dysfunction may play a critical role in the onset and exacerbation of PD‐related non‐motor symptoms. Meanwhile, emerging evidence suggests that glymphatic system dysfunction may also correlate with the progression of idiopathic neuropsychiatric disorders such as anxiety and depression (Y. Zhang et al. 2024; Yang et al. 2024). However, there is currently little research on the relationship between anxiety and glymphatic dysfunction in PD.

Recently, emerging neuroimaging techniques, such as diffusion tensor image analysis along the perivascular space (DTI‐ALPS) and the coupling of global brain function and CSF dynamics (global blood oxygen level‐dependent [gBOLD]‐CSF coupling), have gained traction for evaluating glymphatic function in PD (Si et al. 2022; Cai et al. 2023; Shen et al. 2022; Y. Zhang et al. 2024; Wang et al. 2023) and other neurodegenerative diseases (Yang et al. 2024; Liu et al. 2024; Huang et al. 2024; Han et al. 2024). In comparison to the classic gadolinium‐enhanced glymphatic MRI technique, these emerging neuroimaging techniques have demonstrated robustness and reliability in glymphatic system assessment (W. Zhang. et al. 2021). Therefore, the DTI‐ALPS index and gBOLD‐CSF coupling index coul serve as relative quantitative indicators of brain glymphatic function. Furthermore, combined analysis of these indices may improve the efficiency and precision of brain glymphatic system evaluation (Zhu et al. 2024).

We hypothesized that anxiety in PD is associated with impaired glymphatic system dysfunction, leading to a decreased clearance rate of neurotoxic substances. We further proposed that the DTI‐ALPS index and gBOLD‐CSF coupling could serve as neuroimaging biomarkers for anxiety in PD. This study compared the DTI‐ALPS index and gBOLD‐CSF coupling index among three groups: Patients with PD with anxiety (PD‐A), patients with PD without anxiety (PD‐NA), and healthy controls (HCs). We also evaluated the correlation between the PD anxiety scores and these neuroimaging indices. Finally, we analyzed the diagnostic performance of the DTI‐ALPS and gBOLD‐CSF coupling indices in distinguishing PD‐A from PD‐NA. The purpose of this study is to elucidate the correlation between anxiety and glymphatic dysfunction in PD.

## 2 Methods

### 2.1 Subjects

Seventy patients with PD were recruited from the PD clinic at Affiliated Wuxi People's Hospital of Nanjing Medical University (Wuxi, China) between August 2021 and August 2023. Thirty‐eight HCs were also enrolled for comparison. This study was approved by the Ethics Committee of Affiliated Wuxi People's Hospital of Nanjing Medical University, and all participants provided written informed consent. To minimize the effects of medication, patients discontinued dopamine treatment or taking antipsychotic medicine at least 12 h before undergoing MRI.

The inclusion criteria for patients with PD were as follows: (1) Diagnosis consistent with the Movement Disorder Society (MDS) clinical diagnostic criteria for PD (Postuma et al. 2015); (2) Age > 40 years; (3) Right‐handedness, and (4) voluntary participation in the study. Then, the exclusion criteria for participants were as following: (1) History of severe head trauma or neuropsychiatric disorders; (2) contraindication or inability to undergo MRI; (3) Abnormal findings on routine MRI; (4) Cognitive impairment (MMSE ≤ 26), and (5) Absence of resting state‐functional MRI (rs‐fMRI) imaging slices covering the foramen magnum, which excluded participants from the gBOLD‐CSF coupling analysis or the combined analysis of gBOLD‐CSF coupling and DTI‐ALPS.

### 2.2 Clinical Assessments

Clinical data, including age, sex, education level, PD duration, and levodopa‐equivalent daily dose (LEDD), were collected from patients with PD after the MRI examination. Two experienced neurologists conducted detailed clinical evaluations for each participant, administering the following scales: MDS Unified PD Rating Scale Motor Part 3 (MDS‐UPDRS‐III), Hoehn and Yahr (H&Y) scale, Hamilton Anxiety Rating Scale (HAMA), Hamilton Depression Rating Scale (HAMD), and Mini‐Mental State Examination (MMSE).

Patients were classified into two groups based on their HAMA scores: PD with anxiety (HAMA ≥ 14) and PD without anxiety (HAMA < 14) (Leentjens et al. 2011, Chen et al. 2023).

Similar demographic and clinical data were collected for HCs.

### 2.3 Image Scanning

All participants underwent MRI examinations in the morning using a 3.0T Siemens Prisma MRI system (Siemens, Germany) with a 20‐channel head coil. Foam pads and earplugs were used to minimize the head movement of each subject and reduce noise during image acquisition.

The parameters for three‐dimensional T1‐weighted magnetization‐prepared rapid acquisition gradient echo (3D‐T1 MP‐RAGE) were: repetition time (TR) = 2300 ms, echo time (TE) = 2.98 ms, inversion time (TI) = 900 ms, fractional anisotropy (FA) = 9°, slice thickness = 1 mm, field of view (FOV) = 256 × 256 × 192 mm^3^, matrix size = 256 × 256, voxel resolution = 1 × 1 × 1 mm^3^, and acquisition time (TA) = 5 min 30 s.

The diffusion tensor imaging (DTI) parameters included: TR = 4100 ms, TE = 72 ms, FOV = 210 × 210 mm^2^, diffusion directions = 64, b‐value = 1000 s/mm^2^, slice thickness = 2 mm, no slice gap, voxel size = 2 × 2 × 2 mm^3^, and TA = 5 min 18 s.

For rs‐fMRI, the parameters were: TR = 1500 ms, TE = 31 ms, FA = 70°, FOV = 211 × 211 mm^2^, in‐plane matrix = 64 × 64, slices = 60, slice thickness = 2.4 mm, no slice gap, voxel size = 2.4 × 2.4 × 2.4 mm^3^, time points = 300, TA = 7 min 40 s.

### 2.4 ALPS Analysis

#### 2.4.1 DTI Data Preprocessing

DTI data preprocessing was performed using FMRIB Software Library (FSL) and included the following steps: (1) Phase Encoding Distortion Correction: Geometric distortions due to magnetic field inhomogeneities were corrected using the TOPUP algorithm with posterior‐anterior and anterior‐posterior phase‐encoded images; (2) Eddy Current and Motion Correction: Eddy current distortions and head motion artifacts were corrected using the EDDY algorithm; (3) MP‐PCA denoising: Thermal noise was reduced using Marchenko‐Pastur Principal Component Analysis (MP‐PCA); (4) Gibbs unringing: Gibbs artifacts were removed, improving edge quality; (5) Tensor Calculation: Diffusion tensors were calculated using FSL DTIFIT, generating FA maps and diffusivity maps for each axis (Dxx, Dyy, Dzz); and (6) Normalization: DTI data were registered to the standard John Hopkins University‐International Consortium of Brain Mapping FA (JHU‐ICBM‐FA) template using FLIRT and FNIRT tools in FSL for spatial normalization.

#### 2.4.2 Region of Interest (ROI) Placement

ROIs were defined in the lateral ventricular body, targeting the projection fibers (left and right superior corona radiata [SCR]) and association fibers (left and right superior longitudinal fasciculi [SLF]). Spherical ROIs with a 5 mm diameter were automatically generated in the normalized FA maps based on the JHU‐ICBM‐FA template to encompass the SCR and SLF regions. The standardized coordinates for the ROI centers were: (1) Left SCR (116, 110, 99); (2) Right SCR (64, 110, 99); (3) Left SLF (128, 110, 99); and (4) Right SLF (51, 110, 99).

#### 2.4.3 ALPS Index Calculation

The ALPS index was calculated using the formula:

$$\mathrm{ALPS}\;\mathrm{index}\;=\frac{\mathrm{mean}\left(Dxx_{\mathrm{proj}},Dxx_{\mathrm{assoc}}\right)}{\mathrm{mean}\left(Dyy_{\mathrm{proj}},Dzz_{\mathrm{assoc}}\right)}$$

where (1) Dxx_proj: Diffusivity along the *x*‐axis (left‐right direction) in the projection fiber region; (2) Dxx_assoc: Diffusivity along the *x*‐axis in the association fiber region; (3) Dyy_proj: Diffusivity along the *y*‐axis (anterior‐posterior direction) in the projection fiber region; and (4) Dzz_assoc: Diffusivity along the *z*‐axis (inferior‐superior direction) in the association fiber region.

**FIGURE 1:**
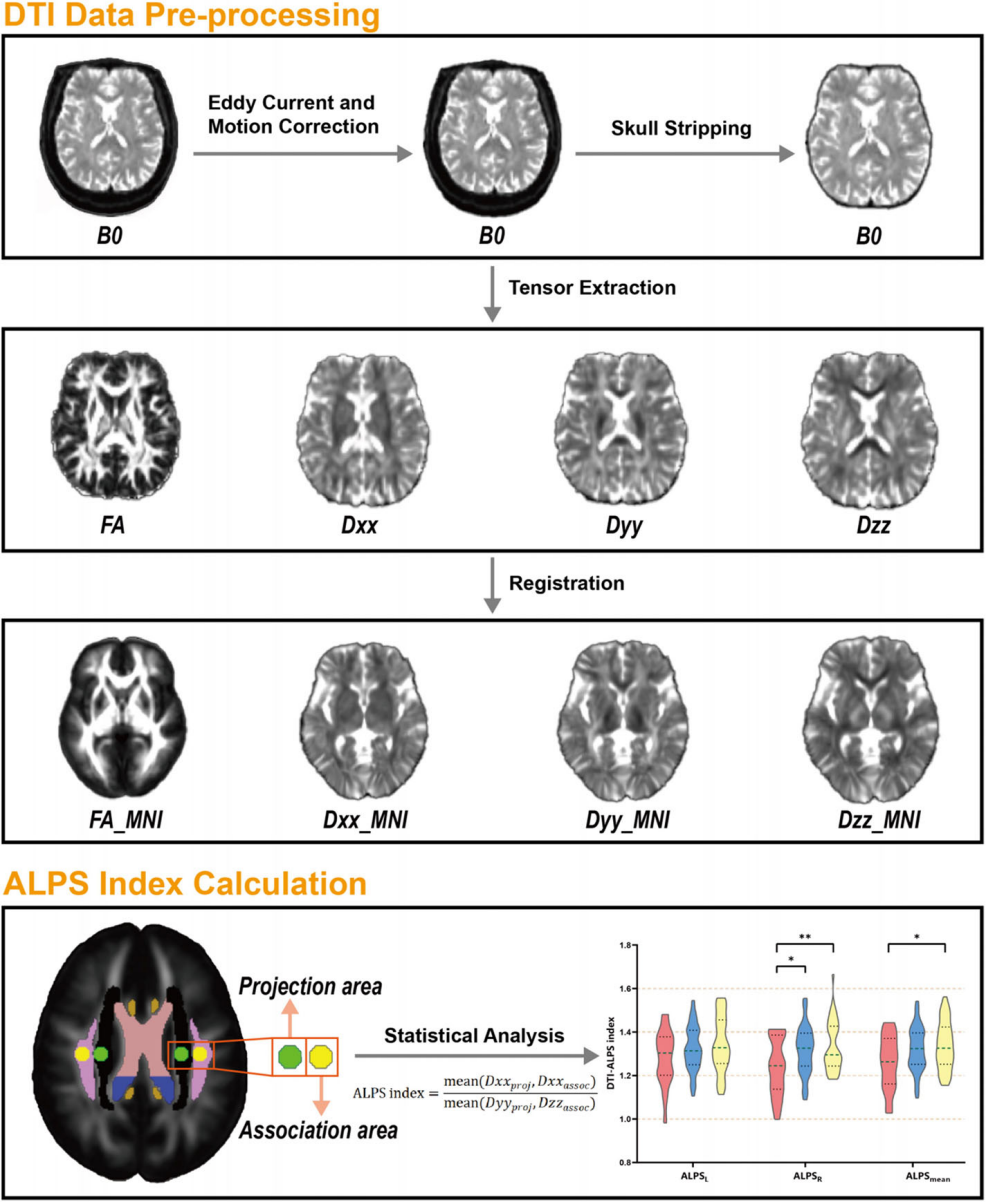
**The process of ALPS analysis**. First, each subject's FA map was calculated and registered into MNI space. Then, four ROIs on association fibers (yellow) and projection fibers (green) in bilateral hemispheres were placed in the FA map in MNI space. Finally, the ALPS index was calculated according to the formula. Abbreviations: ALPS, diffusion tensor image analysis along the perivascular space; Dxx/Dyy/Dzz, diffusitivity map for each axis; FA, fractional anisotropy; MNI, Montreal Neurological Institute; ROI, region of interest.

The process of ALPS analysis is presented in Figure 1.

### 2.5 gBOLD‐CSF Coupling Analysis

#### 2.5.1 rs‐fMRI Data Preprocessing

The rs‐fMRI data were preprocessed using the DPABI toolbox based on SPM12 with the following steps: (1) Removal of the first 10 time points to stabilize magnetization signal stabilization; (2) Slice timing correction and head motion correction; (3) Spatial smoothing with a 6 mm Gaussian kernel; (4) Linear trend removal and bandpass filtering (0.01–0.1 Hz); and (5) Extraction of global gray matter and CSF signals.

#### 2.5.2 Definition of ROI

Whole‐brain gray matter regions were defined using the Harvard‐Oxford cortical structural atlas. The CSF region was identified from the bottom slices of the rs‐fMRI images, ensuring coverage of the foramen magnum to maintain CSF signal stability, and verified using high‐resolution 3D‐T1 images.

#### 2.5.3 Quantification of gBOLD‐CSF Coupling Strength

Gray matter and CSF signals were extracted, and Pearson correlation coefficients were calculated over a time lag range of −5 TR to +5 TR. The gBOLD‐CSF correlation at −3 TR, where the absolute amplitude was highest, was used to quantify coupling strength. Coupling strength was further validated by cross‐correlating the negative derivative signals, with statistical significance determined using a null distribution generated from 10,000 random signal pairings.

**FIGURE 2:**
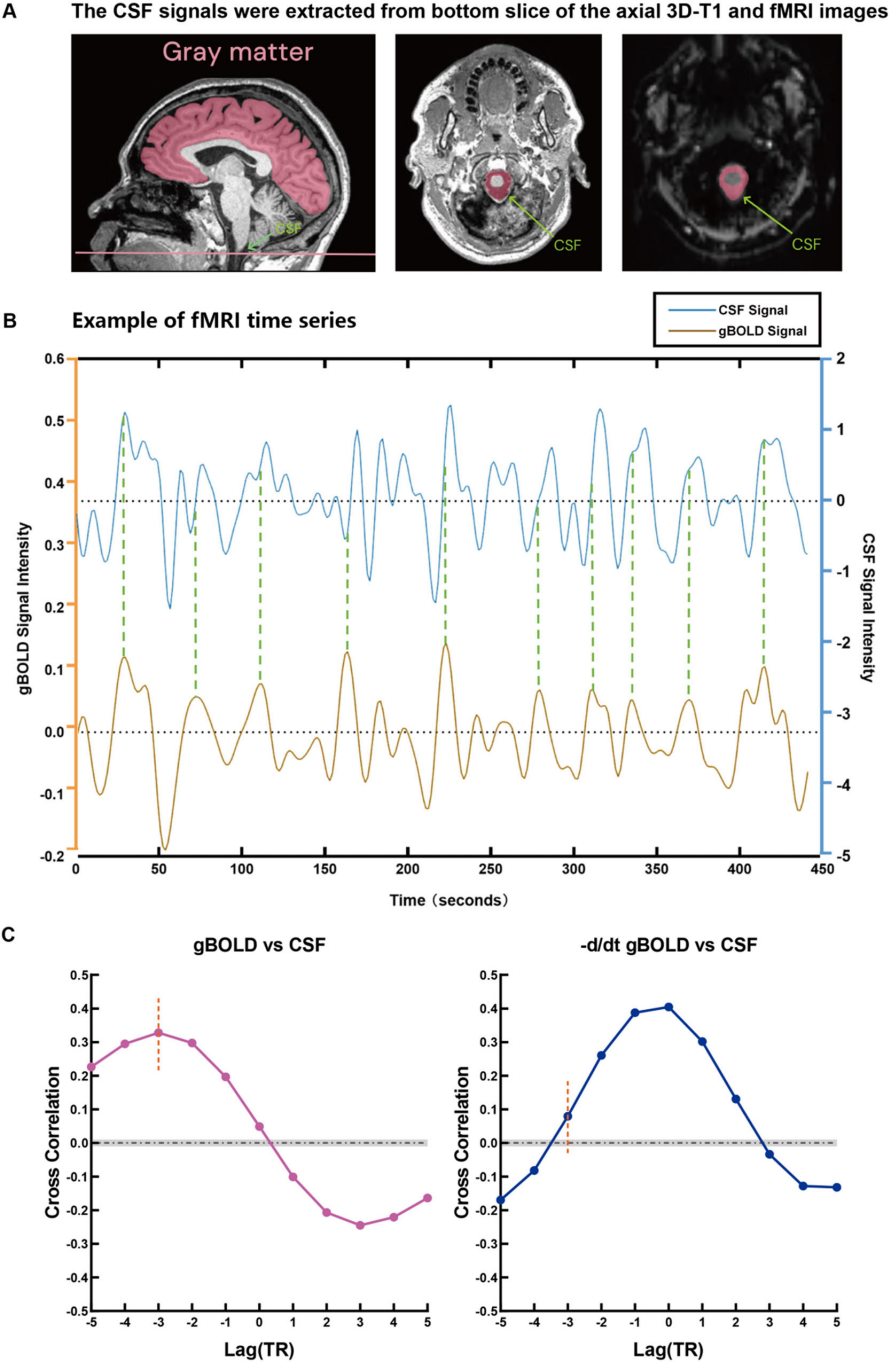
**The process of gBOLD‐CSF coupling analysis. (A)** The CSF signal was extracted from the pseudocolored pink area located at the bottom of the 3D‐T1 and fMRI images. **(B)** Synchronized trends are shown between the gBOLD signal (gold line) and CSF signal (blue line) extracted from a representative example of an fMRI time series, showing CSF signal peaks appeared after the gBOLD signal peaks. **(C)** The mean cross correlation between gBOLD signal and CSF signal was highest at −3 TR. The negative derivative (−dl/dt) of the gBOLD signal was most closely coupled with the CSF signal at 0 TR. Gray dashed lines mark 95% confidence intervals of the mean cross correlation. Abbreviations: CSF, cerebrospinal fluid; fMRI, functional magnetic resonance imaging; gBOLD, global blood oxygen level dependent; TR, repetition time.

The process of gBOLD‐CSF coupling analysis is presented in Figure 2.

### 2.6 Statistical Analysis

Statistical analyses were performed using SPSS version 26.0. Continuous data are expressed as mean ± standard deviation, and categorical data as proportions. After evaluating homoscedasticity and normality, one‐way analysis of variance (ANOVA), two‐sample *t*‐tests, chi‐square tests, and Kruskal–Wallis tests were used to compare the demographic, clinical, and glymphatic characteristics across the PD‐A, PD‐NA, and HC groups. Differences were considered statistically significant at a Bonferroni‐corrected *p* < 0.05. With age, gender, years of education, PD duration, and H&Y stage as covariates, partial correlation analysis was conducted to evaluate relationships between the glymphatic index and anxiety level in patients with PD. After applying the Shapiro–Wilk test for normality, Pearson's correlation analysis was performed to evaluate relationships between the gBOLD‐CSF coupling index and ALPS index in the combined analysis. Receiver operating characteristic (ROC) curve analysis was then used to evaluate the diagnostic performance of clinical features (including PD duration and H&Y stage), the ALPS index, gBOLD‐CSF coupling strength, and a multivariate model for distinguishing PD‐A from PD‐NA. DeLong's test was applied to compare the area under the curve (AUC) of the multivariate model that integrated clinical variables and glymphatic indices with alternative models.

## 3 Results

### 3.1 Population

Two patients with PD were excluded due to poor imaging quality. A total of 68 patients with PD and 38 HCs were included in the ALPS analysis, categorized into the PD‐A (*n* = 21), PD‐NA (*n* = 47), and HC (*n* = 38) groups. For the gBOLD‐CSF coupling analysis, 20 participants were excluded because the bottom slices of the rs‐fMRI images did not include the foramen magnum. Consequently, 52 patients with PD and 34 HCs were included in the gBOLD‐CSF coupling analysis and grouped into PD‐A (*n* = 16), PD‐NA (*n* = 36), and HC (*n* = 34) groups.

### 3.2 ALPS Analysis

#### 3.2.1 Demographic and Clinical Features in the ALPS Analysis

No significant differences were observed in age, sex, education, or MMSE scores among the three groups (*p* > 0.05, Bonferroni‐corrected). Similarly, there were no significant differences between the PD‐A and PD‐NA groups in LEDD, H&Y stage, or UPDRS‐III scores (*p* > 0.05). However, the PD‐A group had a longer disease duration than the PD‐NA group (*p* = 0.016). HAMA and HAMD scores were significantly higher in the PD‐A group than in the PD‐NA and HC groups (*p* < 0.001). No significant differences in HAMA and HAMD scores were found between the PD‐NA and HC groups (*p* > 0.05). The details are presented in Table 1.

**TABLE 1:** Comparison of clinical characteristics and ALPS index among the PD‐A, PD‐NA, and HC groups.

	PD‐A (*n* = 21)	PD‐NA (*n* = 47)	HC (*n* = 38)	Statistics
Gender (M/F)	10/11	30/17	19/19	Pearson's Chi‐Square = 2.315, *p* = 0.314^a^
Age (years)	67.00±7.11	63.00±8.84	62.61±7.99	F = 2.194, *p* = 0.117^b^
Education (years)	10.40±2.19	9.63±3.27	9.55±3.96	H = 0.494, *p* = 0.612^c^
LEDD (mg)	409.52±230.35	409.84±224.58	NA	t = ‐0.005, *p* = 0.996^d^
Disease duration (years)	5.60±3.65	3.36±2.38	NA	t = 2.573, *p* = 0.016^[Link]^, ^[Link]^
H&Y	2.29±0.77	1.93±0.69	NA	t = 1.918, *p* = 0.059^d^
UPDRS‐III	25.24±11.66	21.09±10.66	NA	t = 1.442, *p* = 0.154^d^
HAMA	19.52±4.85	5.43±3.25	2.61±1.76	F = 198.908, *p* < 0.001^[Link]^, ^[Link]^
HAMD	12.43±5.84	4.06±2.83	3.05±1.97	F = 57.300, *p* < 0.001^[Link]^, ^[Link]^
MMSE	28.57±1.69	28.47±1.44	28.97±1.00	H = 1.526, *p* = 0.222^c^
DTI‐ALPS_mean_ (×10^−3^ mm^2^/s)	1.261±0.124	1.321±0.101	1.338±0.110	F = 3.486, *p* = 0.034^[Link]^, ^[Link]^
DTI‐ALPS_L_ (×10^−3^ mm^2^/s)	1.282±0.126	1.324±0.102	1.341±0.128	F = 1.771, *p* = 0.175^b^
DTI‐ALPS_R_ (×10^−3^ mm^2^/s)	1.240±0.131	1.317±0.110	1.334±0.114	F = 4.761, *p* = 0.010^[Link]^, ^[Link]^

Abbreviations: DTI‐ALPSL, left‐hemispheric DTI‐ALPS; DTI‐ALPSR, right‐hemispheric DTI‐ALPS; H&Y, Hoehn & Yahr scales; HAMA, Hamilton Anxiety Rating Scale; HAMD, 17‐item Hamilton Depression Rating Scale; HC, healthy controls; LEDD, levodopa equivalent daily dose; MMSE, Mini‐Mental State Examination; NA, not applicable; PD‐A, Parkinson's disease with anxiety; PD‐NA, Parkinson's disease without anxiety; UPDRS‐III, Unified Parkinson's Disease Rating Scale.

^a^ chi‐square test

^b^one‐way analysis of variance (ANOVA)

^c^kruskal–Wallis test

^d^two‐sample *t*‐test.

^*^Represents a significant difference among the three groups (*p* < 0.05, Bonferroni‐corrected).

#### 3.2.2 ALPS Index

**FIGURE 3:**
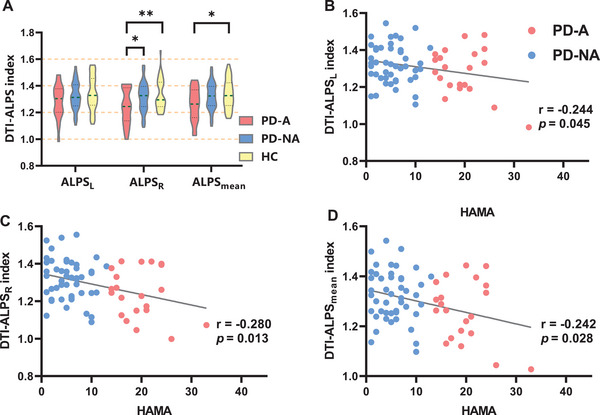
**(A) DTI‐ALPS analysis**. Error bars denote the standard error of the mean. Asterisks denote significant differences between the groups: *Bonferroni‐corrected *p* < 0.05; **Bonferroni‐corrected *p* < 0.01. **(B‐D) DTI‐ALPS correlation analysis**. Significant correlations between ALPS indices and HAMA scores. DTI‐ALPS, diffusion tensor image analysis along the perivascular space; DTI‐ALPS_L_, left ‐hemispheric DTI‐ALPS; DTI‐ALPS_R_, right‐hemispheric DTI‐ALPS; HAMA, Hamilton Anxiety Rating Scale; HAMD, Hamilton Depression Rating Scale; HC, healthy control; PD‐A, Parkinson's disease with anxiety; PD‐NA, Parkinson's disease without anxiety.

The right‐hemispheric ALPS (ALPS_R_) index was significantly lower in the PD‐A group than in the PD‐NA (*p* = 0.014, Bonferroni‐corrected) and HC (*p* = 0.005) groups. In addition, the mean ALPS (ALPS_mean_) index was lower in the PD‐A group than in the PD‐NA (*p* = 0.04) and HC (*p* = 0.017) groups. The details of the ALPS index are presented in Table 1 and Figure 3A.

To control for potential confounding effects of clinical variables, age, gender, years of education, PD duration, and H&Y stage were included as covariates in partial correlation analyses. In patients with PD, the ALPS_L_ (*r* = −0.244; *p* = 0.045), ALPS_R_ (*r* = −0.280; *p* = 0.013), and ALPS_mean_ (*r* = −0.242; *p* = 0.028) indices were negatively correlated with HAMA scores. These relationships are illustrated in Figures 3B–D.

### 3.3 gBOLD‐CSF Coupling Analysis

#### 3.3.1 Demographic and Clinical Features in gBOLD‐CSF Coupling Analysis

No significant differences were found in age, sex, education, or MMSE scores among the three groups (*p* > 0.05, Bonferroni‐corrected). Similarly, there were no significant differences between the PD‐A and PD‐NA groups in LEDD and UPDRS‐III scores (*p* > 0.05). However, the PD‐A group had a longer disease duration (*p* = 0.013) and a higher H&Y stage (*p* = 0.008) than the PD‐NA group. Consistent with the ALPS analysis, the PD‐A group demonstrated significantly higher HAMA and HAMD scores than the PD‐NA and HC groups (*p* < 0.001). Detailed results are presented in Table 2.

**TABLE 2:** Comparison of clinical characteristics, gBOLD‐CSF coupling index and ALPS index among the PD‐A, PD‐NA and HC groups in the combined analysis of gBOLD‐CSF coupling and ALPS.

	PD‐A (*n* = 16)	PD‐NA (*n* = 36)	HC (*n* = 34)	*p* value
Gender (M/F)	8/8	22/14	17/17	Pearson's Chi‐Square = 1.043, *p* = 0.594^a^
Age (years)	66.63 ± 7.63	62.64 ± 9.19	61.35 ± 7.03	F = 2.325, *p* = 0.104^b^
Education (years)	10.66 ± 2.17	9.35 ± 3.40	9.50 ± 3.88	H = 0.866, *p* = 0.424^c^
LEDD (mg)	440.63 ± 220.58	380.90 ± 172.29	NA	t = 1.057, *p* = 0.296^d^
Disease duration (years)	5.84 ± 3.75	3.10 ± 2.17	NA	t = 2.734, *p* = 0.013^d^, ^*^
H&Y	2.34 ± 0.79	1.79 ± 0.60	NA	t = 2.767, *p* = 0.008^d^, ^*^
UPDRS‐III	25.50 ± 12.19	20.36 ± 11.45	NA	t = 1.465, *p* = 0.149^d^
HAMA	18.88 ± 5.06	5.25 ± 3.29	2.68 ± 1.79	F = 143.319, *p* < 0.001^d^, ^*^
HAMD	11.25 ± 4.57	4.03 ± 2.72	3.26 ± 1.96	F = 45.207, *p* < 0.001^d^, ^*^
MMSE	28.75 ± 1.77	28.47 ± 1.38	28.91 ± 1.03	H = 0.958, *p* = 0.388^c^
				
gBOLD‐CSF coupling	0.230 ± 0.143	0.363 ± 0.194	0.337 ± 0.177	F = 3.146, *p* = 0.048^d^, ^*^
DTI‐ALPS_mean_ (×10^−3^ mm^2^/s)	1.256 ± 0.110	1.320 ± 0.105	1.338 ± 0.115	F = 3.061, *p* = 0.05^d^, ^*^
DTI‐ALPS_L_ (×10^−3^ mm^2^/s)	1.282 ± 0.119	1.324 ± 0.105	1.335 ± 0.130	F = 1.124, *p* = 0.330^b^
DTI‐ALPS_R_ (×10^−3^ mm^2^/s)	1.231 ± 0.113	1.317 ± 0.114	1.341 ± 0.119	F = 5.020, *p* = 0.009^d^, ^*^

Abbreviations: DTI‐ALPSL, left‐hemispheric DTI‐ALPS; DTI‐ALPSR, right‐hemispheric DTI‐ALPS; H&Y, Hoehn & Yahr scales; HAMA, Hamilton Anxiety Rating Scale; HAMD, 17‐item Hamilton Depression Ratin g Scale; HC, healthy controls; LEDD, levodopa equivalent daily dose; MMSE, Mini‐Mental State Examination; NA, not applicable; PD‐A, Parkinson's disease with anxiety; PD‐NA, Parkinson's disease without anxiety; UPDRS‐III, Unified Parkinson's Disease Rating Scale.

^a^ chi‐square test

^b^ one‐way analysis of variance (ANOVA)

^c^ kruskal–Wallis test

^d^ two‐sample t‐test.

* Represents a significant difference among the three groups (*p* < 0.05, Bonferroni‐corrected).

#### 3.3.2 gBOLD‐CSF Coupling Index

**FIGURE 4:**
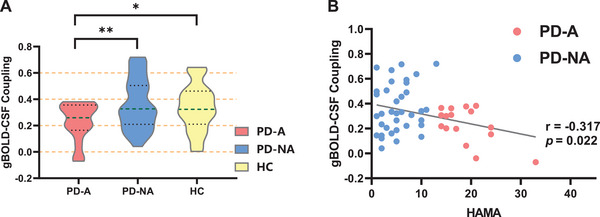
**(A) gBOLD‐CSF coupling analysis**. Error bars denote standard error of the mean. Asterisks denote significant differences between the groups: *Bonferroni‐corrected *p* < 0.05; **Bonferroni‐corrected *p* < 0.01. **(B) gBOLD‐CSF coupling correlation analysis**. Significant correlations between gBOLD‐CSF coupling strength and HAMA scores. gBOLD‐CSF coupling, coupling of global brain function and cerebrospinal fluid dynamics; HAMA, Hamilton Anxiety Rating Scale; HAMD, Hamilton Depression Rating Scale; HC, healthy control; PD‐A, Parkinson's disease with anxiety; PD‐NA, Parkinson's disease without anxiety.

The gBOLD‐CSF coupling strength in the PD‐A group was significantly lower than that in the PD‐NA group (*p* = 0.017, Bonferroni‐corrected) and the HC group (*p* = 0.04). Details are presented in Table 2 and Figure 4A.

With age, gender, years of education, PD duration, and H&Y stage as covariates, the gBOLD‐CSF coupling strength was significantly correlated with HAMA (*r* = −0.317; *p* = 0.022) in patients with PD. These findings are illustrated in Figure 4B.

### 3.4 Combined Analysis of gBOLD‐CSF Coupling and ALPS

#### 3.4.1 Demographic and Clinical Features in Combined Analysis

The demographic and clinical characteristics reported in this section are identical to those presented in Section 3.3.1 (Table 2).

#### 3.4.2 Correlation Between gBOLD‐CSF Coupling Index and ALPS Index

**FIGURE 5:**
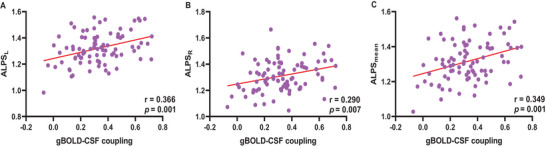
**Correlation analysis between ALPS indices and gBOLD‐CSF coupling strength**. ALPS indices show a significant positive correlation with gBOLD‐CSF coupling strength. DTI‐ALPS, diffusion tensor image analysis along the perivascular space (ALPS_L_, left‐hemispheric DTI‐ALPS; ALPS_R_, right‐hemispheric DTI‐ALPS); gBOLD‐CSF coupling, coupling of global brain function and cerebrospinal fluid dynamics.

A positive correlation was observed between the gBOLD‐CSF coupling strength and all ALPS indices. Specifically, the gBOLD‐CSF coupling index correlated with the ALPS_L_ (*r* = 0.366; *p* = 0.001), ALPS_R_ (*r* = 0.290; *p* = 0.007), and ALPS_mean_ indices (*r* = 0.349; *p* = 0.001). These results are depicted in Figure 5.

#### 3.4.3 The Joint Diagnostic Value of Clinical Features, ALPS Index, and gBOLD‐CSF Coupling Index for Patients With PD‐A

ROC curve analysis was conducted to evaluate the diagnostic performance of statistically significant clinical features (including PD duration and H&Y stage), the ALPS index, gBOLD‐CSF coupling strength, and a combined model incorporating clinical variables and glymphatic indices for differentiating patients with PD‐A from those without. Since the ALPS_mean_ index represents the average of the ALPS_L_ and ALPS_R_ indices, only ALPS_L_ and ALPS_R_ were included in the ROC analysis.

**FIGURE 6:**
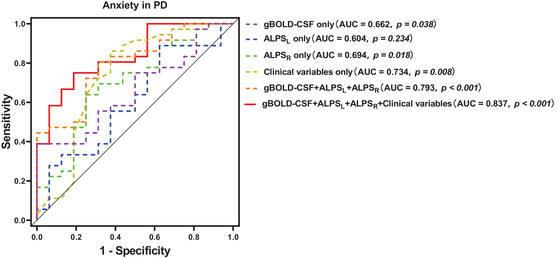
**Model performance for predicting the occurrence of anxiety in PD**. The models utilize the DTI‐ALPS index, gBOLD‐CSF coupling strength, and various clinical variables (including PD duration and H&Y stage) both individually and in combination with glymphatic indices for predicting the occurrence of anxiety in PD. AUC, area under the ROC curve; DTI‐ALPS, diffusion tensor image analysis along the perivascular space (ALPS_L_, left‐hemispheric DTI‐ALPS; ALPS_R_, right‐hemispheric DTI‐ALPS); gBOLD‐CSF coupling, coupling of global brain function and cerebrospinal fluid dynamics; PD, Parkinson's disease; ROC, receiver operating characteristic.

The ALPS_L_ index did not distinguish between patients with PD‐A and those without (*p* > 0.05). However, ALPS_R_, gBOLD‐CSF coupling strength, and clinical variables showed diagnostic potential. The AUC for the model including only gBOLD‐CSF coupling strength was 0.662 (95% confidence interval (CI) = 0.511–0.812, sensitivity = 0.389, specificity = 1). For the model with the ALPS_R_ index alone, the AUC was 0.694 (95% CI = 0.537–0.852, sensitivity = 0.639, specificity = 0.75). The model based solely on clinical variables achieved an AUC of 0.734 (95% CI = 0.563–0.904, sensitivity = 0.625, specificity = 0.861). With all glymphatic indices combined, the model achieved an AUC of 0.793 (95% CI = 0.667–0.920, sensitivity = 0.688, specificity = 0.806). The multivariate model integrating the clinical variables and glymphatic indices demonstrated the highest diagnostic accuracy, with an AUC of 0.837 (95% CI = 0.725–0.949, sensitivity = 0.813, specificity = 0.75). DeLong's test showed that the multivariable model's AUC was significantly higher than that of the models with the gBOLD‐CSF coupling strength alone (0.837 vs. 0.662, *p* < 0.001) or the ALPS_R_ index alone (0.837 vs. 0.694, *p* < 0.001). However, no significant differences were observed when compared to the clinical features‐only model (0.837 vs. 0.734, *p* = 0.110) or the model combining all glymphatic indices (0.837 vs. 0.793, *p* = 0.413), as shown in Figure 6.

## 4 Discussion

To our knowledge, this study is the first to employ both DTI‐ALPS and gBOLD‐CSF coupling analyses to investigate brain glymphatic function alterations among patients with PD‐A, those with PD‐NA, and HCs. The findings reveal that the ALPS index and gBOLD‐CSF coupling strength were significantly reduced in patients with PD‐A compared to patients with PD‐NA and HCs. Both the ALPS index and gBOLD‐CSF coupling strength were inversely correlated with HAMA scores, underscoring their association with anxiety severity in PD. Notably, a positive correlation between the ALPS index and gBOLD‐CSF coupling strength validated the consistency of these MRI methods for quantifying brain glymphatic function. Finally, ROC analysis demonstrated that the model combining clinical variables and glymphatic indices outperformed univariate models in diagnosing anxiety in PD.

Previous studies have established the ALPS index as a reliable marker for assessing brain glymphatic function (Si et al. 2022; Shen et al. 2022; Yang et al. 2024), while gBOLD‐CSF coupling analysis has emerged as a novel tool for glymphatic evaluation in neurodegenerative diseases (Zhang et al. 2024, 2022; Wang et al. 2023). Consistent with earlier findings, our study corroborates findings of significant decreases in the ALPS index in patients with PD (Si et al. 2022; Wang et al. 2023; Gui et al. 2024; Bae et al. 2023). In PD, the pathological accumulation of α‐syn and glymphatic system dysfunction are known to exacerbate one another (Zou et al. 2019; Ding et al. 2021).

Importantly, by integrating two distinct glymphatic function markers, we demonstrated that patients with PD‐A exhibited more severe glymphatic impairment than those without anxiety. This underscores a strong negative relationship between glymphatic dysfunction and anxiety severity in PD, which may be due to anxiety associated with PD increasing the burden on the glymphatic system. As is widely recognized, severe anxiety is frequently accompanied by significant sleep disturbances (Nicholson and Pfeiffer 2021). This correlation has also been consistently observed in patients with PD, where both anxiety and sleep disorders rank among the most prevalent non‐motor symptoms (Thorpy and Adler 2005; He et al. 2024). Research has shown that sufficient sleep facilitates the clearance of metabolic waste products from the brain and enhances glymphatic system function (Xie et al. 2013). In PD, multiple neuroimaging studies have further demonstrated a close association between glymphatic dysfunction and sleep disturbances (Nepozitek et al. 2025; Scott‐Massey et al. 2022; Meinhold et al. 2025). Therefore, we hypothesized that anxiety may impair glymphatic function through the mediating effect of sleep disorders. Unfortunately, sleep‐related data were not collected from the PD cohort in this study. Consequently, the interpretation that anxiety affects glymphatic function from this perspective necessitated proof through future studies.

In addition, abnormalities in cerebral aquaporin‐4 (AQP4) water channel protein in PD may also represent one of the underlying reasons for the strong correlation between anxiety and glymphatic system dysfunction. The AQP4 channel facilitated bidirectional water exchange between CSF and the interstitial fluid, and abnormalities in this channel were likely a key cause of cerebral glymphatic dysfunction related to PD (Iliff et al. 2012; Sun et al. 2023; Lapshina and Ekimova 2024). While animal studies have demonstrated that modulating AQP4 can influence anxiety‐related behaviors in other neurological conditions (Nie et al. 2024), it is important to note that the direct evidence linking AQP4 specifically to anxiety in PD remains limited. Therefore, AQP4‐related glymphatic dysfunction may represent one potential mechanism among others. Together with our findings, this body of evidence suggests that glymphatic system impairment could be a contributing factor to the high comorbidity of anxiety in PD, though the exact mechanisms warrant further investigation.

Both ALPS and gBOLD‐CSF coupling are valuable MRI indicators for evaluating the glymphatic system, but because different MRI sequences are required for these analyses, few studies have employed both methods simultaneously (Zhu et al. 2024). While the robustness of ALPS in glymphatic function assessment had been validated through comparisons with traditional gadolinium‐enhanced MRI methods (W. Zhang. et al. 2021), gBOLD‐CSF coupling analysis required further validation. The consistency observed between the ALPS index and gBOLD‐CSF coupling strength in this study indirectly supports the reliability of gBOLD‐CSF coupling analysis for evaluating brain glymphatic function.

The results of the ROC analysis in this study demonstrated that the combined model of the ALPS index and gBOLD‐CSF coupling strength significantly improved the diagnostic performance for detecting anxiety in patients with PD compared to their respective univariate models. Although these two MRI glymphatic methods evaluate similar indicators, they appear to complement each other in the diagnosis of clinical conditions. These findings suggest that glymphatic MRI markers could help stratify patients with PD according to their risk of developing anxiety.

This study has some limitations: (1) It was a single center study with a relatively small sample size; (2) some subjects were excluded from the joint DTI‐ALPS analysis due to fMRI data that did not meet the post‐processing requirements for gBOLD‐CSF coupling analysis; (3) the study design was cross‐sectional, although follow‐up data collection is ongoing; (4) the influence of comorbid depressive symptoms in PD could not be entirely excluded, possibly due to a significant overlap in scoring items between the HAMA and HAMD scales (Chen et al. 2023); (5) the study employed a limited set of scales for evaluating anxiety symptoms in PD; and (6) the study did not collect sleep assessment information to investigate the relationship among anxiety, sleep, and glymphatic function. Future research will incorporate additional scales to enable a more precise stratification of patients with PD with anxiety.

In conclusion, the findings from DTI‐ALPS and gBOLD‐CSF coupling analyses support the hypothesis that anxiety in PD is closely associated with glymphatic dysfunction. The combined model of glymphatic indicators and clinical features significantly improved the diagnostic accuracy for detecting anxiety in PD. Both DTI‐ALPS and gBOLD‐CSF coupling show potential as neuroimaging markers for identifying anxiety in PD, but the complex interactive mechanisms among anxiety, the glymphatic system, and PD remain to be unraveled.

## Author Contributions

KC, BH, and XF: Conceptualization. KC, LZ, FW, YJ, LL, and RZ: Data curation. KC and BH: Formal analysis. XF: Funding acquisition. KC: Investigation. BH: Methodology. XF: Project administration. XF and FW: Resources. BH: Software. XF: Supervision. KC and BH: Validation. KC and BH: Visualization. KC: Writing—original draft. KC, LL, LZ, XH, and XF: Writing—review & editing. All authors contributed to this study and approved the submitted version.

## Ethics Statement

The ethical application of this study has been reviewed and approved by the Ethics Committee of Wuxi People's Hospital affiliated to Nanjing Medical University.

## Consent

Participants involved in this study all provided their written informed consent.

## Conflicts of Interest

The authors declare no conflicts of interest.

## Peer Review

The peer review history for this article is available at https://publons.com/publon/10.1002/brb3.70918

## Data Availability

The raw data will be made available by the corresponding authors without reservation.
